# Maternal Antiretroviral Therapy for the Prevention of Mother-To-Child Transmission of HIV in Malawi: Maternal and Infant Outcomes Two Years after Delivery

**DOI:** 10.1371/journal.pone.0068950

**Published:** 2013-07-19

**Authors:** Marina Giuliano, Mauro Andreotti, Giuseppe Liotta, Haswell Jere, Jean-Baptiste Sagno, Martin Maulidi, Sandro Mancinelli, Ersilia Buonomo, Paola Scarcella, Maria F. Pirillo, Roberta Amici, Susanna Ceffa, Stefano Vella, Leonardo Palombi, Maria Cristina Marazzi

**Affiliations:** 1 Department of Therapeutic Research and Medicines Evaluation, Istituto Superiore di Sanità, Rome, Italy; 2 Department of Biomedicine and Prevention, University of Tor Vergata, Rome, Italy; 3 DREAM Program, Community of S. Egidio, Rome, Italy; 4 DREAM Program, Community of S. Egidio, Blantyre, Malawi; 5 DREAM Program, Community of S. Egidio, Lilongwe, Malawi; 6 LUMSA University, Rome, Italy University, Rome, Italy; University of Washington, United States of America

## Abstract

**Background:**

Optimized preventive strategies are needed to reach the objective of eliminating pediatric AIDS. This study aimed to define the determinants of residual HIV transmission in the context of maternal antiretroviral therapy (ART) administration to pregnant women, to assess infant safety of this strategy, and to evaluate its impact on maternal disease.

**Methodology/Principal Findings:**

A total of 311 HIV-infected pregnant women were enrolled in Malawi in an observational study and received a nevirapine-based regimen from week 25 of gestation until 6 months after delivery (end of breastfeeding period) if their CD4+ count was > 350/mm^3^ at baseline (n = 147), or indefinitely if they met the criteria for treatment (n. 164). Mother/child pairs were followed until 2 years after delivery. The Kaplan-Meier method was used to estimate HIV transmission, maternal disease progression, and survival at 24 months. The rate of HIV infant infection was 3.2% [95% confidence intervals (CI) 1.0-5.4]. Six of the 8 transmissions occurred among mothers with baseline CD4+ count > 350/mm^3^. HIV-free survival of children was 85.8% (95% CI 81.4-90.1). Children born to mothers with baseline CD4+ count < 350/mm^3^ were at increased risk of death (hazard ratio 2.6, 95% CI 1.1-6.1). Among women who had stopped treatment the risk of progression to CD4+ count < 350/mm^3^ was 20.6% (95% CI 9.2-31.9) by 18 months of drug discontinuation.

**Conclusions:**

HIV transmission in this cohort was rare however, it occurred in a significative proportion among women with high CD4+ counts. Strategies to improve treatment adherence should be implemented to further reduce HIV transmission. Mortality in the uninfected exposed children was the major determinant of HIV-free survival and was associated to maternal disease stage. Given the considerable proportion of women reaching the criteria for treatment within 18 months of drug discontinuation, life-long ART administration to HIV-infected women should be considered.

## Introduction

Administration of antiretroviral therapy (ART) to HIV-infected women during pregnancy and, postpartum, during breastfeeding, is one of the recommended options for the prevention of HIV mother-to-child transmission of the latest World Health Organization (WHO) guidelines, issued in 2010 [1]. Efficacy of this strategy has been clearly demonstrated in randomized and observational studies [2–7]. However, although cumulative rates of infection at 12-18 months are significantly reduced, HIV transmission still occurs in a small proportion of the exposed infants. With the objective of eliminating pediatric HIV infection, understanding the determinants of this residual transmission is crucial.

Recently, different resource-limited countries have also expressed significant interest in a possible new strategy for HIV-infected women (Option B-plus) : the administration of life-long antiretroviral therapy, irrespective of the CD4+ count, to all pregnant women [8]. This strategy has actually been implemented in Malawi since July 2011. Potential advantages include the harmonization of treatment and prevention programmes, avoiding stopping and starting of antiretroviral drugs, and prevention of sexual transmission. In this context further data informing this policy can be relevant.

In the present study we analyzed the data of a cohort of HIV-infected pregnant women receiving ART for the prevention of mother-to-infant transmission and of their children followed up until 24 months after delivery. We had the following objectives : a) to identify the determinants of residual transmission in this setting; b) to evaluate the safety in the exposed infants; c) to evaluate the safety in the mothers and to assess their immunological and virological status 24 months after delivery; d) to determine the proportion, among those discontinuing ART, who had to resume treatment because of a new pregnancy or a low CD4+ count e) to identify the determinants of the loss to follow-up.

## Methods

### Ethics Statement

The study was approved by the National Health Sciences Research Committee of Malawi (approval number 486). A separate informed consent was signed by all participants.

### Study population and setting

Study population included HIV-positive pregnant women in Malawi attending two Ante Natal Clinics (one in Blantyre and one in Lilongwe) of the DREAM (Drug Resource Enhancement and Malnutrition) Program, designed and managed by the Community of S. Egidio, an Italian faith-based non-governmental organization. Women older than 16, naïve to antiretrovirals (with the exception of single-dose nevirapine), willing to breastfeed up to 6 months (the recommended duration of breastfeeding at the time of the study [9]), with no grade 3 or 4 laboratory toxicity and no active tuberculosis were enrolled in an observational study aimed to assess the pharmacokinetics and the safety of administration of ART to lactating women [SMAC (Safe Milk for African Children) study]. Data on determination of drug levels have been previously published [10].

### Clinical Procedures

ART was started as soon as possible after the first trimester for women meeting the criteria for treatment according to the DREAM program [7] (CD4+ count < 350/mm^3^). For the other women, prophylaxis was started at week 25 of gestation age or as soon as possible if they presented after week 25. Women with CD4+ count < 350/mm^3^, received a combination of stavudine (d4T, 30 mg twice daily), lamivudine (3TC, 150 mg twice daily) and nevirapine (NVP, 200 mg twice daily) as first-line recommended therapy in Malawi at the time, and continued the same treatment after the end of breastfeeding. Women with a CD4+ count > 350/mm^3^, received a regimen of zidovudine (ZDV, 300 mg twice daily), lamivudine and nevirapine (the preferred strategy used by the DREAM program) until 6 months after delivery or longer if breastfeeding was not stopped at 6 months. Women experiencing nevirapine-associated toxicity were allowed to replace this drug with either indinavir (IDV, 800 mg three times a day) or lopinavir-ritonavir (LPV/r, 400/100 mg twice daily). All infants received a single-dose of NVP syrup (2mg/kg of body weight) within 72 h of birth. Women were instructed to exclusively breastfeed for 4.5 months and then wean their infants over a 1.5 month period, until complete cessation of breastfeeding at 6 months. At each visit after Month 6 information was collected about possible continuation of breastfeeding, and the presence of breast milk was verified by manual breast expression.

### Laboratory Procedures

Hemato-chemistry and immunological laboratory testing was performed at the local DREAM laboratories in Malawi. Laboratory abnormalities were classified with the use of the Division of AIDS Toxicity Tables [11]. Infants’ infection status was assessed locally at 1, 3, 6, and 12 months using a qualititative HIV-1 DNA PCR assay (Amplicor HIV-1 DNA v 1.5 assay, Roche, Branchburg, NJ, USA). At 18 months an enzyme-linked immunosorbent assay (ELISA) test was performed. An infant was considered HIV infected based on a positive HIV-DNA PCR assay at any visit, and a positive ELISA at month 18.

Viral load in plasma and breast milk samples collected up to 6 months was measured at the laboratory of the Istituto Superiore di Sanità, Rome, using the kPCR Versant assay (Siemens Healthcare Diagnostics, Deerfield, IL, USA) with a lower limit of 37 copies/ml. HIV-RNA determination in plasma samples collected after 6 months was performed in Malawi using the Versant bDNA assay (Siemens Healthcare Diagnostics) with a lower limit of detection of 50 copies/ml.

### Statistical analysis

Descriptive data are presented as means with standard deviations (SD) and medians with Inter Quartile Ranges (IQR). Differences between means were tested with the Student *t* test and differences between medians with the Mann-Whitney test. Categorical data were compared using the chi-square test or the Fisher test, as appropriate.

The Kaplan-Meier method was used to estimate the rates of loss to follow-up, of HIV transmission and of mother and child survival (including HIV-free survival). The log-rank test was used to compare groups defined by the maternal CD4+ count at baseline (> or < 350/mm^3^). In case of twins only the first-born was included in the transmission and survival analyses. Children lost to follow-up were censored at the date of their last negative HIV test for analyses of transmission and of HIV-free survival and at the date of last visit for mortality analysis. Time of HIV infection was estimated as the midpoint between the date of the last negative sample and the date of the first positive test. For the analysis of late postnatal transmission earlier infections were censored at the time of first HIV positive test. The Cox proportional hazards model was used to identify factors associated with mortality in children and loss to follow-up. Variables with P values < 0.2 in univariate analysis were included as covariates in the final multivariate models. For all statistical tests, two-sided P values of less than 0.05 were considered to be statistically significant. Data analysis was performed using the SPSS software system 20.0 (IBM, Somers, NY, USA).

## Results

### Study participants characteristics

**Figure 1:**
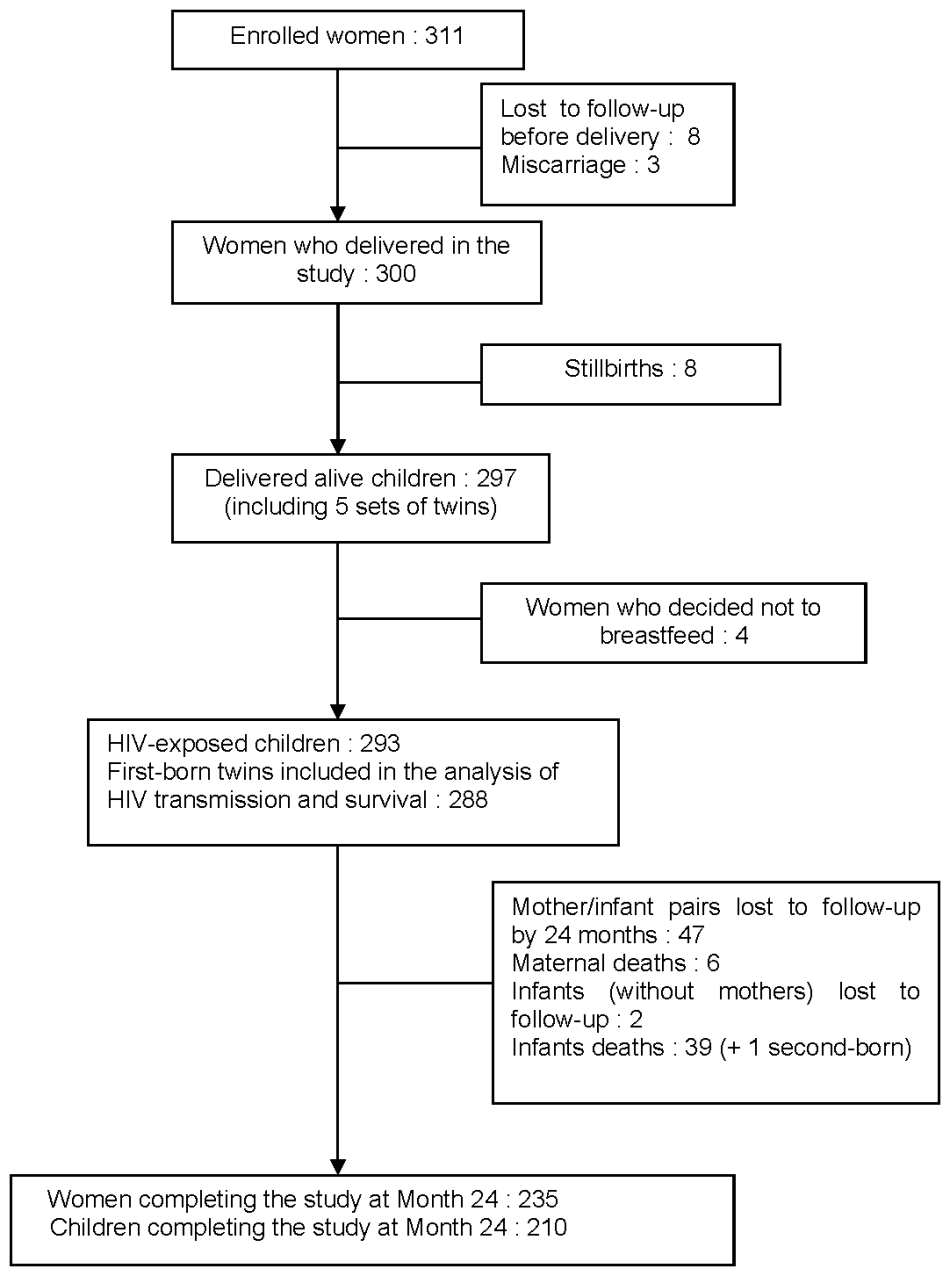
Cohort profile.

A total of 311 women were enrolled between February 2008 and February 2009 (Figure 1). Characteristics of the enrolled women and of the children of the study are reported in Table 1.

**Table 1:** Characteristics of women and children in the study.

			**All**	**Baseline CD4^+^ count > 350/mm^3^**	**Baseline CD4^+^ count < 350/mm^3^**
**Women**			311	147	164
	***Age***, median (IQR)		27 (23-30)	26 (23-29)	28 (24-32)
	***WHO Stage***, n (%)	I	230 (74.4%)	119 (81.0%)	111 (67.7%)
		II	56 (18.1%)	22 (15.0%)	34 (20.7%)
		III	22 (7.1%)	4 (2.7%)	18 (11%)
		IV	1 (0.3%)	-	1 (0.6%)
	***Previous use of sdNVP***, n (%)	Yes	16 (5.1%)	12 (8.2%)	4 (2.4%)
		No	190 (61.1%)	83 (56.5%)	107 (65.2%)
		Unknown	105 (33.7%)	52 (35.4%)	53 (32.3%)
	***% of women with ≥ 1 previous dead child***		21.4%	14.9%	27.2%
	***Weekofgestationatscreening***, median (IQR)		24 (20-28)	24 (21-29)	23 (19-28)
	***CD4+ count/mm*** ^3^, median (IQR)		339 (214-492)	503 (427-614)	218 (138-301)
	***ViralLoad* (*logcopies*)**, median (IQR)		4.1 (3.3-4.6)	3.7 (2.9-4.3)	4.4 (3.9-4.8)
	***% of women with VL* >50,000 copies/ml**		20.6%	10.9%	29.3%
	***Hemoglobin***, median (IQR)		10.3 (9.4-11.2)	10.5 (9.6-11.4)	10.2 (9.1-11)
	***Week of gestation at ART initiation***, median (IQR)		26 (24-30)	26 (25-30)	25 (22-29)
	***Initial triple ARV regimen***, n (%)	ZDV+3TC+NVP	146 (46.9%)	143 (97.3%)	3^a^ (1.8%)
		d4T+3TC+NVP	165 (53.1%)	4^b^ (2.7%)	161 (98.2%)
	***Viral load at delivery (N. 96)***, median (IQR)		37 (37-130)	37 (37-104.5)	54 (37-212)
	***% of women with VL* < 400 copies/ml at delivery**		88/96 (91.7%)	65/69 (94.2%)	23/27 (85.2%)
	***Durationofbreastfeeding* (*weeks*)**, median (IQR)		25.7 (25.4-26.4)	25.7 (25.4-26)	25.7 (25.4-26.3)
	** *N. breastfeeding* *after6months***, (%)^c^		66 (22.2%)	25 (17%)	41 (25%)
					
**Children**			297	142	155
	***Gender*^*d*^**	Female	158 (53.9%)	78 (54.9%)	80 (51.6%)
		Male	135 (46.1%)	62 (43.7%)	73 (47.1%)
	***Placeofbirth***	Health Center	176 (59.8%)	81 (57%)	95 (61.3%)
		Hospital	96 (32.7%)	47 (33.1%)	49 (31.6%)
		Home	22 (7.5%)	14 (9.9%)	8 (5.2%)
	***Typeofdelivery***	Vaginal Delivery	263 (91.0%)	123 (86.6%)	140 (90.3%)
		Delivery with episiotomy	10 (3.5%)	5 (3.5%)	5 (3.2%)
		Cesarean section	16 (5.5%)	9 (6.3%)	7 (4.5%)
	***NVPdoseatbirth***	Yes	248 (84.4%)	118 (83.1%)	130 (83.9%)
		No	46 (15.6%)	24 (16.9%)	22 (14.2%)
	***ARTduringpregnancy* (*days*)**, median(IQR)		71 (45-95)	63 (40-85)	75 (52-106)
	***Birthweight*^*e*^ (g)**, median (IQR)		3.2 (2.7-3.5)	3.2 (2.7-3.5)	3.2 (2.65-3.5)
		Low birth weight (< 2500 g)	35/250 (14%)	14/121 (11.6%)	21/155 (16.3%)
		Very low birth weight (< 1500 g)	2/250 (0.8%)	1/121 (0.8%)	1/155 (0.8%)

^a^ Physician’s decision;^b^ Anemia in 3 cases, physician’s decision in one case; ^c^ Either presence of breast milk or reported breastfeeding after month 6 ^d^ For 4 infants who died soon after delivery the gender is not known; ^e^ Data on 250 newborns. Weight measured either at birth or within 15 days of birth

At baseline women had a median CD4+ count of 339/mm^3^ and an HIV-RNA level of 4.1 log_10_ copies/ml. Median duration of breastfeeding was 25.7 weeks but, for 22.2% of the women, there was evidence of breastfeeding beyond 6 months (either presence of breast milk or reported breastfeeding after Month 6 visit). No evidence of breastfeeding was found after Month 15.

### Mother-to-child transmission

Final HIV status was available for 278/288 (96.5%) children included in the analysis of transmission; among them 8 (4 males and 4 females) acquired the infection. All were delivered vaginally and received sd-NVP at birth. The cumulative risk of HIV infection was 3.2% (95% CI 1.0-5.4) by 24 months. Two infants first tested positive 1 month post-partum (either in utero, intrapartum or early breastfeeding transmissions), 2 during the period of breastfeeding (one at Month 3 and one at Month 6) and 4 at Month 12 (all 4 were HIV-negative at Month 6) (Table 2). Although a baseline CD4+ count > 350/mm^3^ was not significantly associated with the transmission risk (P = 0.12) it has to be noted that 6 of the 8 (75%) transmissions occurred among women with baseline CD4+ cell count above this threshold (in 3 cases > 500/mm^3^). Only one transmitting woman (N. 3) had previously been exposed to sd-NVP. In 1 of the 2 women who transmitted during the expected duration of breastfeeding (N. 4) the HIV-RNA levels were high both in plasma and in breast milk at the time preceding the first detection of HIV in the infant. Among the women who transmitted after 6 months two (N. 5 and 6) were on continuous therapy. The risk of late postnatal transmission (occurring after 6 months) was higher (P = 0.013, by the log-rank test), in women who had evidence of breastfeeding beyond Month 6.

**Table 2:** Maternal and infant characteristics of the cases of HIV transmission.

	**Maternal data** **CD4+ count/mm^*3*^**	**Plasma HIV-RNA (copies/ml)**	**Breast Milk HIV-RNA (copies/ml)**	**Infant data HIV-DNA test**	**ART duration before birth (days)**
**N. 1**					40
Enrolment	649	19,700			
Month 1	578	67	<37	+	
**N. 2**					28
Enrolment	730	201			
Month 1	NA	NA		+	
**N. 3**					97
Enrolment	462	132,000			
Month 1	NA	<37	<37	-	
Month 3	514	<37	90	+	
**N. 4**					86
Enrolment	723	55,709			
Month 1	NA	12,452	8,753	-	
Month 3	NA	NA	NA	-	
Month 6	880	668	<37	+	
**N. 5**					81
Enrolment	68	153,688			
Month 1	113	44	101	-	
Month 3	133	43	<37	-	
Month 6	224	<37	41	-	
Month 12°	265	422	-	+	
**N. 6**					56
Enrolment	124	8,664			
Month 1	253	<37	293	-	
Month 3	NA	<37	<37	-	
Month 6#	261	<37	<37	-	
Month 12	331	<37	-	+	
**N. 7**					87
Enrolment	430	NA			
Month 1	NA	<37	<37	-	
Month 3	205	<37	<37	-	
Month 6#	617	<37	<37	-	
Month 12*	194	305,000	-	+	
**N. 8**					31
Enrolment	413	87,477			
Month 1	NA	84	NA	-	
Month 3	714	<37	NA	-	
Month 6	597	<37	NA	-	
Month 12*	455	8,694	-	+	

NA = Not available

All women were on treatment at all time-points with the exception of those with * (drugs discontinued 6 months after delivery in women with baseline CD4+ count above 350/mm^3^).

° Woman reporting having breastfed until Month 15

# Presence of breast milk until Month 9 for woman N. 6 and until Month 7 for woman N. 7, respectively.

### Child survival and HIV-free survival

**Figure 2:**
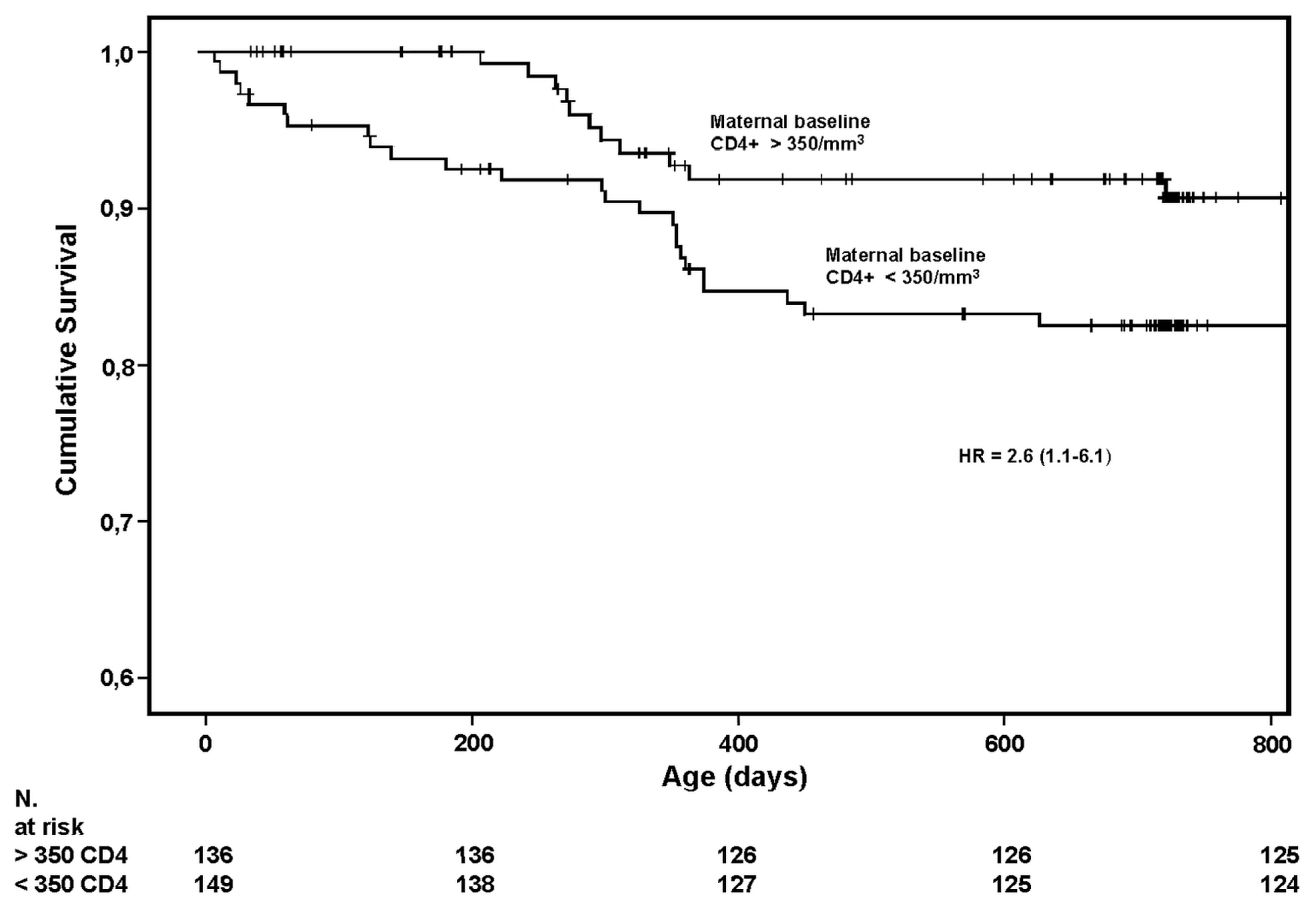
Kaplan-Meier child survival analysis according to baseline maternal CD4+ count (above or below 350/mm^3^).

Thirty-nine children (2 HIV-infected) died during follow-up. Neonatal mortality was 2.1% (6/288). Excluding the 3 infants who died within 24 hours (never breastfed), the cumulative survival rate was 90% (95% CI 86.4-93.5) at 12 months and 86.3% (95% CI 82.1-90.4) at 24 months. In univariate analysis maternal CD4+ count < 350/mm^3^ (P = 0.043), and a lower birth weight (P = 0.074) were associated to child mortality. In a multivariate model (including 243 children with available birth weight out of the total 288), adjusting for maternal baseline hemoglobin level, week of gestation at screening, and the presence of previous dead children, only maternal CD4+ count < 350/mm^3^ remained associated [hazard ratio 2.6 (95% CI 1.1-6.1), P = 0.02] (Figure 2). Main causes of deaths were gastroenteritis (n. 12), pneumonia (n. 7), malaria (n. 7) and malnutrition (n. 4) (Table 3). HIV-free survival was 86.6% (95% CI 82.4-90.7) at 12 months and 85.8% (95% CI 81.4-90.1) at 24 months. HIV-free survival was not significantly different between children born to mothers with baseline CD4+ count above or below 350/mm^3^ (P = 0.36).

**Table 3:** Summarized causes of death in children (excluding neonatal deaths).

	**1-6 months**	**7-12 months**	**13-24 months**	**Total**	**HIV-positive**
Gastroenteritis	3	7	2	12	0
Pneumonia	1	5	1	7	1
Malaria	2	2	3	7	0
Measles	0	1	0	1	0
Malnutrition	0	1	3	4	1
Unknown	1	1	0	2	0
**Total**	7	17	9	33	2

### Maternal viro-immunological outcomes and survival

**Figure 3:**
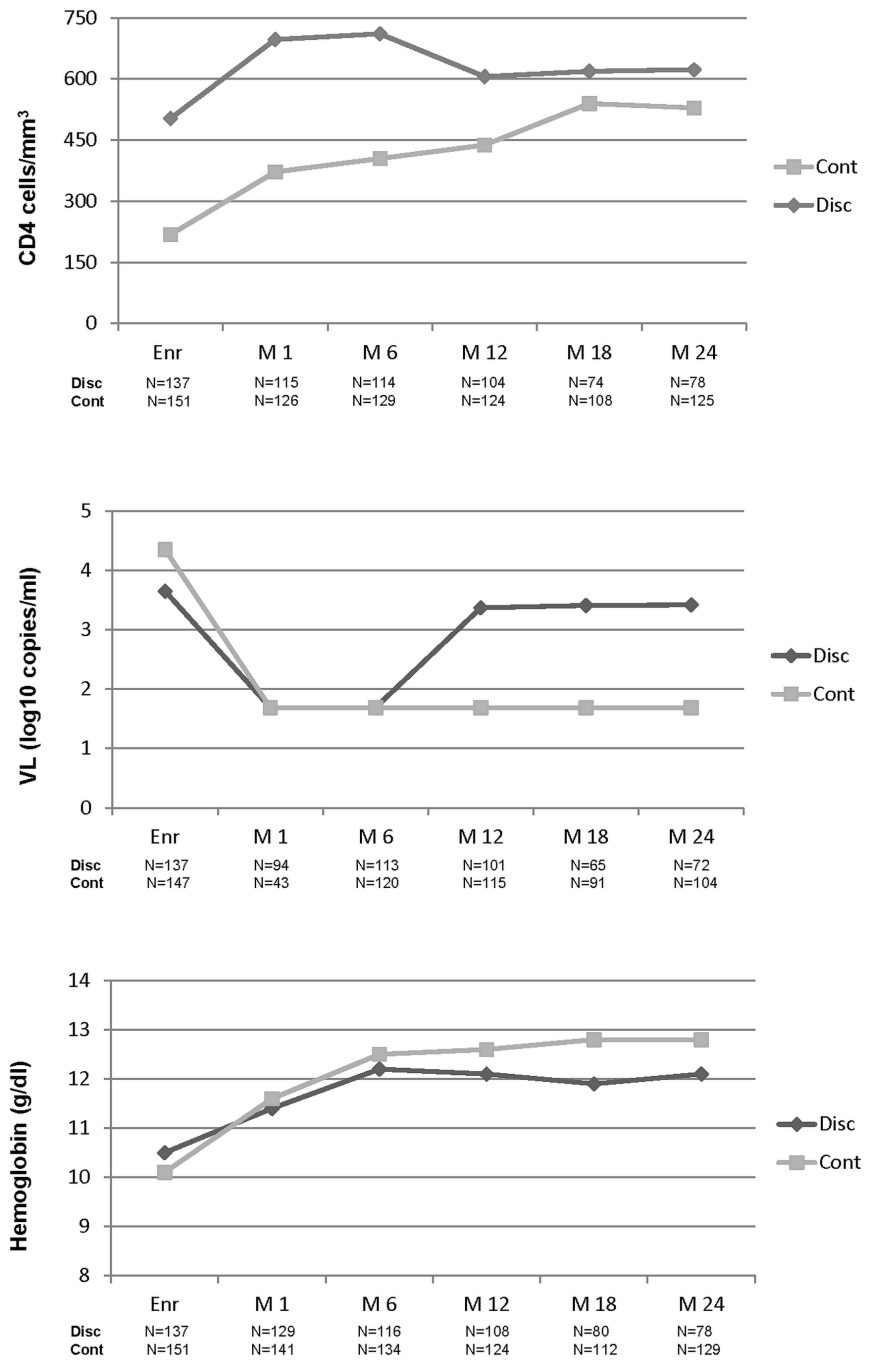
Longitudinal assessment of CD4+ count (median cells/mm^3^), viral load (median log_10_ copies/ml) and hemoglobin levels (median g/dl) in women in continuous therapy (Cont.) and in those who had discontinued treatment 6 months postpartum (Disc.). Analysis conducted on the 288 women who had an alive child and breastfed. Number of determinations available are reported for each timepoint. Data of women in the discontinuing group who resumed ART were included until treatment re-initiation.

Figure 3 reports the longitudinal assessment of maternal viro-immunological parameters and hemoglobin levels according to the maternal CD4+ group at baseline. Among the women on continuous therapy (baseline CD4+ count < 350/mm^3^) there was a progressive increase of the CD4+ count until Month 24, while, for women discontinuing drugs 6 months after partum, a progressive although slow decrease was observed between Month 6 and Month 24 (from a median of 711 cells/mm^3^ to a median of 623 cells/mm^3^). In both groups > 85% of women had HIV-RNA < 400 copies/ml at Month 6 (86.7% in the group with baseline CD4+ count > 350/mm^3^ and 91.7% in the group with < 350/mm^3^, P = 0.22). At Month 24 HIV replication was still suppressed in a high proportion of women in the group on continuous therapy (84.6% had HIV-RNA < 400 copies/ml compared to 20.8% in the group who had discontinued drugs at 6 months). Women on continuous therapy had a higher hemoglobin level at Month 24 compared to the women who had discontinued drugs (P = 0.001).

Six women died at a median time of 4.9 months (IQR 3.3-10.3) after treatment initiation, 2 of them within 42 days of delivery. Maternal mortality rate was 0.7% (95% CI 0.2-1.6) and cumulative mortality at 2 years 2.1% (95% CI 0.53-1.56). Only 1 of the 6 women had a CD4+ count > 350/mm^3^ at baseline (P = 0.14 for survival in women with baseline count > or < 350/mm^3^). Causes of maternal death were tuberculosis in 2 cases, Kaposi sarcoma, pneumonia, malaria and complications of delivery in one case each. Among the baseline factors only age was significantly different between the women remaining alive and those who died (mean age was 27 years in those who survived vs 23 years in those who died, respectively, P = 0.047). Occurrence of severe (grade 3/4) anemia was significantly more frequent in the mothers who died compared to those remaining alive : 3/6 (50%) vs 26/305 (8.5%), P = 0.012.

### Adverse events in women and children

A total of 29 women (9.3%) developed a grade 3 or 4 anemia after a mean time of 11.2 weeks (SD 15.8) (in 16 cases during pregnancy); 12 were on treatment with ZDV and 17 with d4T (P = 0.52). A grade 3 or 4 skin rash presented in 15 women (4.8%) at a mean time of 3.9 weeks (SD 1.1) after treatment initiation. There was no significant difference in the proportion of women with severe rash according to the baseline CD4+ count threshold of 350/mm^3^. Seven women developed severe liver toxicity (grade 3 or 4) (2.25%) : 5/147 (3.4%) women with baseline CD4+ < 350/mm^3^ vs 2/164 (1.2%) women with baseline CD4+ > 350/mm^3^(P = 0.26). Overall, nevirapine-associated toxicity (including grade 3 or 4 events and assuming that all liver toxicity was related to nevirapine) occurred in 22/311 women (7.1%) (10.2% with baseline CD4+ count < 350/mm^3^ vs 4.2% women with baseline CD4+ > 350/mm^3^, P = 0.048). Thirty-four women (10.9%) modified their treatment because of adverse events, 19 because of NVP-associated toxicity (6.1%), (in 3 cases for increased levels of liver enzymes (1.0%) and in 16 cases for skin rashes (5.1%) including a grade 2 skin rash) (Table 4).

**Table 4:** Adverse Events among women and children by 24 months.

	**Absolute Number (%)**	**Number of women requiring an ARV substitution**	**ARV substituted**
**Mothers (N. 311)**			
Anemia (Grade 3/4)	29 (9.3%)	9	Zidovudine
Skin rash (Grade 3/4)	15 (4.8%)	15	Nevirapine
ALT or AST increase (Grade 3/4)	7 (2.3%)	3	Nevirapine
Thrombocytopenia (Grade 3/4)	4 (1.3%)		
Severe polyneuropathy	3 (0.9%)	3	Stavudine
Lactic acidosis	1 (0.3%)	1	Stavudine
Diarrhea (Grade 3/4)	5 (1.6%)		
**Children (N. 293 exposed children)**			
Anemia (Grade 3/4)	109 (37.2%)		
Skin rash (Grade ≥ 2)	17 (5.8%)		
ALT or AST increase (Grade 3/4)	3 (1.2%)		
Thrombocytopenia (Grade 3/4)	4 (1.4%)		
Diarrhea (Grade ≥ 2)	71 (24.2%)		

A total of 109 children developed a grade 3 or 4 anemia during follow-up (109/293 exposed children = 37.2%), 50 of the 138 (36.2%) children born from mothers on ZDV and 59 of the 155 (38.1%) d4T-exposed (P = 0.81). Of those developing anemia by 6 months (n. 72) 35 were exposed to ZDV and 37 exposed to d4T (P = 0.78). Development of anemia in children was associated with a lower hemoglobin maternal level at baseline (10.1 g/dl in those developing anemia vs 10.4 g/dl, P = 0.016), and a lower infant weight at Month 1 (3.9 Kg in those developing anemia vs 4.1 Kg, P = 0.009). A total of 71 children had an episode of grade ≥ 2 diarrhea and in 12 cases the gastroenteritis contributed to death. The frequency of diarrheal morbidity was rare before month 3 (2.0%), was highest from month 6 with weaning process (17.1% between 6 and 12 months) and declined after month 12 (3.4% between 12 and 24 months). Out of the 17 cases of grade ≥ 2 skin rash, 9 occurred within the first 6 months and 8 after the end of breastfeeding. Among the 18 cases of grade ≥ 2 liver toxicity, 10 occurred during the first 6 months. None of these child events was correlated with the maternal CD4+ cell count group at baseline (> or < 350/mm^3^).

### Post-partum ART re-initiation

Before the end of follow-up 45 of the 292 (15.4%) women who delivered an alive child initiated a second pregnancy. Among them 20 had a CD4+ count at baseline > 350/mm^3^ and had discontinued treatment 6 months after delivery. One of them had a miscarriage, 17 resumed treatment (3 of them had also a CD4+ count < 350/mm^3^ at the time of the new pregnancy), while two had not yet re-started treatment before the end of the study. A further 10 women had to resume treatment because of CD4+ count < 350/mm^3^ and therefore meeting the criteria for treatment. The probability of re-starting ART (either because of a new pregnancy or because of a low CD4+ count) was 29.8% (95% CI 15.6-43.9) by 18 months of drug discontinuation. The probability of progression to a CD4+ count less than 350/mm^3^ for women who had reached 6 months postpartum and discontinued ART (126) was 5.9% (95% CI 1.7-10.0%) 1 year after delivery and 20.6% (95% CI 9.2-31.9%) at 24 months. In this analysis all women who had at least one determination of CD4+ count of less than 350/mm^3^ (n. 19) were included even if they had not restarted treatment before the end of the study. There were no deaths in this group of women. Only one woman among those who had immunological progression had a CD4+ count > 500/mm^3^ at baseline. Women who progressed had a significantly lower median baseline CD4+ count compared to those who did not progress (416/mm^3^ vs 562/mm^3^ P < 0.001) and significantly lower CD4+ count at 6 months (531/mm^3^ versus 772/mm^3^, P < 0.001). Baseline viral load, age, baseline weight, duration of ART during pregnancy, duration of breastfeeding, HIV-RNA at 6 months were not significantly different.

### Loss to follow-up

Among the 292 women who delivered an alive child, 47 were lost before 24 months (18 before 6 months post-partum, and 29 after). The probability of loss to follow-up was 16.4% (95% CI 12.1-20.7%) at 2 years. In univariate analysis both a baseline CD4+ cell count > 350/mm^3^ (P = 0.002) and a baseline hemoglobin level above the median (P = 0.006) were predictors of the loss to follow-up. In a multivariate model, adjusting for age and week of gestation at ART initiation, both the baseline CD4+ count group (hazard ratio 2.2, 95% CI 1.1-4.2, P = 0.019) and a higher baseline hemoglobin (hazard ratio 2.3, 95% CI 1.2-4.4, P = 0.009) remained significantly associated. The probability of loss to follow-up after 6 months postpartum was 10.8% (95% CI 7.0-14.5). The same factors were predictors of the loss to follow-up after 6 months (P = 0.025 for baseline CD4+ count > 350/mm^3^ and P = 0.038 for baseline hemoglobin level above the median).

## Discussion

The Joint United Nations Programme on HIV/AIDS (UNAIDS) has proposed the ambitious goal of the elimination of HIV infections in children, keeping their mothers alive, by 2015 [12]. Implementation of known effective strategies is the priority to meet this goal. However, more information on the safety and effectiveness of the preventive regimens can help in defining optimized strategies. In our study maternal ART administration was significantly beneficial for women’s health (with a very low mortality rate) and highly effective, with a transmission rate similar to other observational and randomized studies [2,13,14]. Although the very limited number of infections did not allow us to establish a significant correlation between one or more factors and the overall risk of transmission, some of our findings were intriguing. First, 6 out of the 8 transmission cases occurred in women with baseline CD4 > 350/mm^3^ (and in 3 cases > 500/mm^3^), underlining the importance of providing maximally efficacious strategies also to the women in less advanced disease stages. Also, in our study half of the cases of HIV transmission occurred between 6 and 12 months, a time when almost all mothers had reported they had weaned their child. Although we cannot exclude that some transmissions may have occurred at the very end of the expected duration of breastfeeding, continued breastfeeding beyond 6 months was the most likely mechanism of these infections (evidence of longer breastfeeding was indeed significantly associated to late infections), and the extension of the period of breastfeeding (and of prophylaxis) up to 1 year in the latest World Health Organization guidelines [1] represents a major step in this sense. However, it has to be noted that 2 of the 4 late transmitting women were on continuous therapy. Moreover, there was evidence in one further case (the woman who had high viral load in plasma and breast milk at Month 1) that drugs were not taken correctly, supporting the idea of the crucial role of treatment adherence in preventing transmission.

Actually, a recent review [15] including > 20,000 women in high- and low-income countries, reported that only 73% of pregnant women were strictly adherent to the preventive regimens (75.7% during pregnancy and only 53% during the postpartum period), suggesting that actions to address the issue of drug adherence need to be taken while the preventive regimens are widely implemented.

The infant safety of these regimens [2–6] was confirmed in our study. No specific toxicity could be attributed to the use of these maternal drugs. Emergence of severe anemia was the most common adverse event although no correlation was found with zidovudine exposure, as previously reported by our group [10]. The development of severe anemia in children was correlated to a lower maternal hemoglobin level at baseline and to a lower infant weight at Month 1 suggesting that a major role has to be attributed to the high prevalence of malnutrition in the country [16,17].

Child mortality was still significant and similar to other contemporary cohorts in Malawi [18,19]. The high rate of gastroenteritis associated to early weaning [20,21] has certainly played a role, further supporting the prolonged period of breastfeeding of the new guidelines. Indeed, in the context of maternal ART administration, HIV transmission seemed to have a relatively low impact on HIV-free survival while mortality of the HIV-exposed uninfected infants remained the major determinant. Interestingly, in our study the only predictor of child mortality was maternal baseline CD4+ count < 350/mm^3^ (this was not true for HIV-free survival, due to the significant proportion of transmitting women with high CD4+ count). In a previous study conducted in Malawi in the pre-ART era [19], infant birth weight represented the most important factor associated to mortality up to 2 years. We cannot exclude the role of birth weight in our study since it was of borderline significance in univariate analysis and because this parameter was not available for all the children. However, it seems that a healthier condition of the mother, as indicated by a higher CD4+ count, could have a significant impact on child survival, as in previous reports [22–24]. Indeed, it has been hypothesized that reduced transplancental transfer of IgG antibodies and immunogical deficiencies in breast milk could play a role [25–27]. Since all women with baseline CD4+ < 350/mm^3^ were on continuous therapy, it seems that effective treatment initiated in the last trimester of pregnancy would not be enough to reverse the risk associated with advanced maternal disease. Long-term treatment of HIV-infected women in resource-limited countries would be helpful in this regard.

One of the most relevant findings of our study was that 30% of the women who had discontinued treatment 6 months postpartum had to resume it (either because of a new pregnancy or a low CD4+ count) by 24 months and that one in five women reached the CD4+ count criteria for treatment by 18 months of drug discontinuation. High fertility rate is common in resource-limited countries, and in the case of drug discontinuation after delivery it would be associated to repeated cycles of “starting and stopping” ART. Significant rates of progression were also reported in a secondary analysis of the Kesho-Bora trial [28] : 43/169 (25.4%) women discontinuing drugs at 6 months reached a CD4+ count < 350/mm^3^ within 2 years, compared to 19/126 (15.1%) (with a Kaplan-Meier estimate of progression of 20.6%) in our study. Although the two study populations were similar, the longer duration of treatment during and after delivery in our study (6 and 18.7 median weeks in Kesho-Bora and 10 and 25 weeks in our study, respectively) may explain the observed difference.

Our findings on the immunological and virological data at 24 months extends data obtained in a previous study at 1 year [29], showing the declining trend in the CD4+ count and the expected rebound of viral load in women discontinuing ART. Interestingly, in our study hemoglobin levels at Month 24 remained lower in women discontinuing drugs at 6 months compared to women who did not stop the drugs, who indeed continued to benefit from drug administration.

Loss to follow-up in our study was associated with higher hemoglobin levels and higher CD4+ counts at baseline. It seems therefore that a relatively good health status could not favour retention in life-long programmes, as suggested in a recent report [30]; this issue should be carefully addressed in designing Option B-plus implementation. Another possible explanation is that women with a baseline CD4+ count > 350/mm^3^, who were scheduled to discontinue drugs 6 months after delivery, were less motivated to return regularly to the clinic. Future studies of early ART will determine if treatment administration provides a stronger link to care.

Our study had several limitations including the observational nature, the relatively low number of women and children included, the lack of complete information on hospitalization and the clinical adverse events, and on drug adherence. Also, since HIV test was not performed at birth, some in utero transmissions could have been unrecognized and therefore responsible, to some extent, for

early mortality. However, our results underline the high efficacy of this strategy, provide indication of possible areas of improvement, and support with some evidences the expansion of universal ART to all HIV-infected pregnant women.

## References

[1] World Health Organization Antiretroviral drugs for treating pregnant women and preventing HIV infection in infants : recommendations for a public health approach. 2010 Version. Available: http://www.who.int/hiv/pub/mtct/antiretroviral2010/en/index.html. Accessed: February 12, 2013. 26180894

[2] The Kesho Bora Study Group (2011) Triple antiretroviral compared with zidovudine and single-dose nevirapine prophylaxis during pregnancy and breastfeeding for prevention of mother-to-child transmssion of HIV-1 (Kesho Bora study): a randomised controlled trial. Lancet Infect Dis 11: 1171-1180. 10.1016/S1473-3099(10)70288-7 21237718

[3] Chasela CS, Hudgens MG, Jamieson DJ, Kayira D, Hosseinipour MC et al. (2010) Maternal or infant antiretroviral drugs to reduce HIV-1 transmission. N Engl J Med 362: 2271-2281. doi:10.1056/NEJMoa0911486. PubMed: 20554982. 20554982 10.1056/NEJMoa0911486 PMC3440865

[4] Shapiro RL, Hughes MD, Ogwu A, Kitch D, Lockman S et al. (2010) Antiretroviral regimens in pregnancy and breast-feeding in Botswana. N Engl J Med 362: 2282-2294. doi:10.1056/NEJMoa0907736. PubMed: 20554983. 20554983 10.1056/NEJMoa0907736 PMC2999916

[5] Thomas T, Masaba R, Borkowf CB, Ndivo R, Zeh C et al. (2011) Triple-antiretroviral prophylaxis to prevent mother-to-child HIV transmission through breastfeeding – The Kisumu Breastfeeding Study, Kenya: a clinical trial. PLOS Med 8(3): e1001015. 21468300 10.1371/journal.pmed.1001015 PMC3066129

[6] Kilewo C, Karlsson K, Ngarina M, Massawe A, Lyamuya E et al. (2009) Prevention of mother-to-child transmission of HIV-1 through breastfeeding by treating mothers with triple antiretroviral therapy in Dar es Salaam, Tanzania: The Mitra Plus Study. J Acquir Immune Defic Syndr 52: 406-416. doi:10.1097/QAI.0b013e3181b323ff. PubMed: 19730269. 19730269 10.1097/QAI.0b013e3181b323ff

[7] Palombi L, Marazzi MC, Voetberg A, Magid NA (2007) Treatment acceleration program and the experience of the DREAM program in prevention of mother-to-child transmission of HIV. AIDS 21: S65-S71. doi:10.1097/01.aids.0000279708.09180.f5. PubMed: 17620755. 10.1097/01.aids.0000279708.09180.f5 17620755

[8] World Health Organization 4 2012) Use of antietroviral drugs for treating pregnant women and preventing HIV infection in infants. Programmatic update. Available: http:// www.who.int/hiv/pub/mtct/programmatic_update2012/en/index.html. Accessed: February 12, 2013. 26180894

[9] World Health Organization (25-27 10 2006) HIV and infant feeding : update based on the technical consultation held on behalf of the Inter-Agency Team (IATT) on Prevention of HIV Infections in Pregnant Women, Mothers and their Infants, Geneva. Available: http://apps.who.int/iris/bitstream/10665/43747/1/9789241595964_eng.pdf. Accessed: April 24, 2013.

[10] Palombi L, Pirillo MF, Andreotti M, Liotta G, Erba F et al. (2012) Antiretroviral prophylaxis for breastfeeding transmission in Malawi: drug concentrations, virological efficacy and safety. Antivir Ther 17: 1511-1519. doi:10.3851/IMP2315. PubMed: 22910456. 22910456 10.3851/IMP2315

[11] Division of AIDS (2004) Division of AIDS Table for Grading the Severity of Adult and Pediatric Adverse Events, version 1.0. National Institute of Health. Available: http://rsc.tech-res.com/document/safetyandpharmacovigilance/Table_for_Grading_Severity_of_Adult_Pediatric_Adverse_Events.pdf. Accessed: February 12, 2013.

[12] UNAIDS. (2011) Global Plan towards the elimination of new infections among children by 2015 and keeping their mothers alive. Joint United Nations Programme on HIV/AIDS.

[13] Dryden-Peterson S, Jayeoba O, Hughes MD, Jibril H, Keapoletswe K et al. (2011) Highly active antiretroviral therapy for prevention of mother-to-child transmission in a programmatic setting, Botswana. J Acquir Immune Defic Syndr 58: 353-357. doi:10.1097/QAI.0b013e31822d4063. PubMed: 21792062. 21792062 10.1097/QAI.0b013e31822d4063 PMC3196679

[14] Gartland MG, Chintu NT, Li MS, Lembalemba MK, Mulenga SN et al. (2013) Field effectiveness of combination antiretroviral prophylaxis for the prevention of mother-to-child HIV transmission in rural Zambia. AIDS 27; Jan 16 (Epub ahead of print). PubMed: 23324656. 10.1097/QAD.0b013e32835e3937 PMC3836017 23324656

[15] Nachega JB, Uthman OA, Anderson J, Peltzer K, Wampold S et al. (2012) Adherence to antiretroviral therapy during and after pregnancy in low-, middle and high income countries: a systematic review and meta-analysis. AIDS 26: 2039-2052. doi:10.1097/QAD.0b013e328359590f. PubMed: 22951634. 22951634 10.1097/QAD.0b013e328359590f PMC5061936

[16] Aiga H, Matsuoka S, Kuroiwa C, Yamamoto S (2009) Malnutrition among children in rural Malawian fish-farming households. Trans R Soc Trop Med Hyg 103: 827-833. doi:10.1016/j.trstmh.2009.03.028. PubMed: 19409590. 19409590 10.1016/j.trstmh.2009.03.028

[17] Maleta K, Virtanen SM, Espo M, Kulmala T, Ashorn P (2003) Childhood malnutrition and its predictors in rural Malawi. Paediatr Perinat Epidemiol 17: 384-390. doi:10.1046/j.1365-3016.2003.00519.x. PubMed: 14629321. 14629321 10.1046/j.1365-3016.2003.00519.x

[18] Taha TE, Li Q, Hoover DR, Mipando L, Nkanaunena K et al. (2011) Postexposure prophylaxis of breastfeeding HIV-exposed infants with antiretroviral drugs to age 14 weeks: updated efficacy results of the PEPI-Malawi trial. J Acquir Immune Defic Syndr 57: 319-3255. doi:10.1097/QAI.0b013e318217877a. PubMed: 21423025. 21423025 10.1097/QAI.0b013e318217877a

[19] Taha TE, Dadabhai SS, Sun J, Rahman MH, Kumwenda J, Kumwenda N (2012) Child mortality levels and trends by HIV status in Blantyre, Malawi: 1989-2009. J Acquir Immune Defic Syndr 60: 462-465. doi:10.1097/QAI.0b013e31825ddcfa. PubMed: 22614899. 22692091 10.1097/QAI.0b013e3182618eea PMC3458133

[20] Taha TE, Hoover DR, Chen S, Kumwenda NI, Mipando L et al. (2011) Effects of cessation of breastfeeding in HIV-1-exposed, uninfected children in Malawi. Clin Infect Dis 53: 388-395. doi:10.1093/cid/cir413. PubMed: 21810754. 21810754 10.1093/cid/cir413 PMC3202326

[21] Fawzy A, Arpadi S, Kankasa C, Sinkala M, Mwiya M, Thea DM (2011) Early weaning increases diarrhea morbidity and mortality among uninfected children born to HIV-infected mothers in Zambia. J Infect Dis 203: 1222-1230. doi:10.1093/infdis/jir019. PubMed: 21459815. 21459815 10.1093/infdis/jir019 PMC3069726

[22] Kuhn L, Kasonde P, Sinkala M, Kankasa C, Semrau K et al. (2005) Does severity of HIV diseases in HIV-infected mothers affect mortality and morbidity among their uninfected infants? Clin Infect Dis 41: 1654-1661. doi:10.1086/498029. PubMed: 16267740. 16267740 10.1086/498029 PMC1351118

[23] Chilongozi D, Wang L, Brown L, Taha T, Valentine M et al. (2008) Morbidity and mortality among a cohort of human immunodeficiency virus type 1-infected and uninfected pregnant women and their infants from Malawi, Zambia, and Tanzania. Pediatr Infect Dis 27: 808-814. doi:10.1097/INF.0b013e31817109a4. PubMed: 18679152. 10.1097/INF.0b013e31817109a4 PMC2739309 18679152

[24] Becquet R, Marston M, Dabis F, Moulton LH, Gray G et al. (2012) Children who acquire HIV infection perinatally are at higher risk of early death than those acquiring infection through breastmilk: a meta-analysis. PLOS ONE 7: e28510. doi:10.1371/journal.pone.0028510. PubMed: 22383946. 22383946 10.1371/journal.pone.0028510 PMC3285615

[25] Moraes-Pinto MI, Almeida AC, Kenj G, Filgueiras TE, Tobias W et al. (1996) Placental transfer and maternally acquired neonatal IgG immunity in human immunodeficiency virus infection. J Infect Dis 173: 1077-1084. doi:10.1093/infdis/173.5.1077. PubMed: 8627057. 8627057 10.1093/infdis/173.5.1077

[26] Jones CE, Naidoo S, De Beer C, Esser M, Kampmann B, Hesseling AC (2011) Maternal HIV infection and antibody responses against vaccine-preventable diseases in uninfected infants. JAMA 305: 576-584. doi:10.1001/jama.2011.100. PubMed: 21304083. 21304083 10.1001/jama.2011.100

[27] Thomas JE, Bunn JEG, Kleanthous H, Monath TP, Harding M et al. (2004) Specific immunoglobulin A antibodies in maternal milk and delayed *Helicobacter pylori* colonization in Gambian infants. Clin Infect Dis 39: 1155-1160. doi:10.1086/424514. PubMed: 15486839. 15486839 10.1086/424514

[28] the Kesho Bora Study Group (2012) Maternal HIV-1 disease progression 18-24 months postdelivery according to antiretroviral prophylaxis regimen (triple-antiretroviral prophylaxis during pregnancy and breastfeeding vs zidovudine/single-dose nevirapine prophylaxis): The Kesho Bora randomized controlled trial. Clin Infect Dis 55: 449-460. doi:10.1093/cid/cis461. PubMed: 22573845. 22573845 10.1093/cid/cis461 PMC3393708

[29] Pilotto JH, Velasque LS, Friedman RK, Moreira RI, Veloso VG et al. (2011) Maternal outcomes after HAART for the prevention of mother-to-child transmission in HIV-infected women in Brazil. Antivir Ther 16: 349-356. doi:10.3851/IMP1779. PubMed: 21555817. 21555817 10.3851/IMP1779 PMC3437753

[30] Alamo ST, Colebunders R, Ouma J, Sunday P, Wagner G et al. (2012) Return to normal life after AIDS as a reason for lost to follow-up in a community-based antiretroviral treatment program. J Acquir Immune Defic Syndr 60: e36-e45. doi:10.1097/FTD.0b013e3182526e6a. PubMed: 22622076. 22622076 10.1097/FTD.0b013e3182526e6a PMC3872063

